# Correlation between Functional Group and Formation of Nanoparticles in PEBAX/Ag Salt/Al Salt Complexes for Olefin Separation

**DOI:** 10.3390/polym12030667

**Published:** 2020-03-17

**Authors:** So Young Kim, Younghyun Cho, Sang Wook Kang

**Affiliations:** 1 Department of Chemistry, Sangmyung University, Seoul 03016, Korea; rlathdud625@naver.com; 2 Department of Energy Systems Engineering, Soonchunhyang University, Asan 31538, Korea; 3 Department of Chemistry and Energy Engineering, Sangmyung University, Seoul 03016, Korea

**Keywords:** olefin, paraffin, copolymer, facilitated transport, nanoparticles

## Abstract

poly ether-block-amide (PEBAX)-2533/metal salt/Al salt membranes were prepared for mixed olefin/paraffin separation. PEBAX-2533 with 80% ether group and 20% amide group was suggested as the polymer matrix for comparison of separation performance according to the functional group ratio in copolymer PEBAX. In addition, Al salts were used to stabilize metal ions for a long time as additives. High permeance was expected with the proportion of high ether groups, since these functional groups provided relatively permeable regions. As a result, the PEBAX-2533 composite membrane showed a selectivity of 5 (propylene/propane) with 10 GPU. However, the permeance of membrane was not unexpectedly improved and the selectivity was reduced. The result was analyzed by using SEM, RAMAN and thermogravimetric analysis (TGA), including Fourier transform infrared (FTIR). The reduction in separation performance was determined by using FT-IR. Based on these results, in order to stabilize the metal ions interacting with the polymer through Al(NO_3_)_3_, it was concluded that a specific ratio of the amide group was needed in PEBAX as a polymer matrix.

## 1. Introduction

Olefins are one of the important raw materials in the petroleum industry [1]. Olefins have been acquired by using the Fischer-Tropsch reaction of coal or the catalytic cracking of petroleum [2,3]. Light olefins have been commonly produced together with the corresponding paraffins, but extremely high purity olefins (>99.9%) have been required to produce polymers. Therefore, olefin/paraffin separation has been such an important process in the petrochemical industry [4,5]. The olefin/paraffin separation has been accomplished nowadays by using cryogenic distillation [6,7,8]. However, distillation processes demand large amounts of energy and equipment costs due to the chemical similarity between the vapor pressure of olefin and paraffin gas [9,10].

In order to save huge costs in the olefin separation process, several separation methods have been proposed in recent years [11]. Several attempts have been made to develop various separation methods such as hybrid membrane-distillation, adsorption, absorption and membranes [12,13]. Hybrid membrane-distillation aims to remove C2 splitters or C3 splitters by installing parallel membrane modules with distillation columns [11]. Adsorptive separation utilizes a method in which one component is selectively adsorbed to an adsorbent particle layer and the other component is passed through. The adsorbed components could later be recovered by either temperature swing adsorption (TSA) or pressure swing adsorption (PSA) [12]. Adsorbents including zeolite 5A, NaX and highly porous MIL-100(Fe) were investigated for their olefin/paraffin separation performance [14,15]. The membrane technology has offered the advantages of low energy requirements, required capital investment, installation space and operating costs [4]. In particular, polymer membranes have become an attractive alternative to traditional separation methods because of several advantages such as good mechanical stability, low cost and ease of processing. [4,12]. The polymer membrane technology was used not only for olefin/paraffin separation but also for various gas separation processes such as using natural gas containing CO_2_ CH_4_ [16,17,18,19]. This technology was being researched in various fields such as MMM technology using ZIF, PIM and facilitated transportation technology using carriers.

Among these fields, the membrane method using the facilitated transport concept has been increasingly utilized. [10,20,21,22]. Ag ions could coordinate reversibly with olefins such as propylene and were known as an effective olefin carrier [23,24]. However, Ag ions were readily reduced to metal nanoparticles (NPs), while generated NPs are deactivation and have the disadvantage of acting as an active barrier [25]. In our group, studies were performed using Al(NO_3_)_3_ to prevent the reduction of Ag ions. [26,27]. The performance of specific polymer/AgBF_4_/Al(NO_3_)_3_ complex membrane was maintained for 14 days, and the white color of the membrane remained for 3 months, indicating the stable metal ions [26]. Furthermore, a polymer with hydroxyl groups/AgBF_4_/Al(NO_3_)_3_ and a complex membrane showed the propylene/propane selectivity of 17 and a mixed gas permeance of 11 GPU (1 GPU = 1 × 10^−6^ cm^3^ (STP)/(cm^2^ s cmHg)) for 145 h [27]. 

On the other hand, poly ether-block-amide (PEBAX) was known as a thermoplastic elastomer with good physicochemical stability and has obtained interest due to its permeable property towards various gas molecules [28]. To compare the permeance performance according to the monomer ratio of copolymer, we used two types of PEBAX-1657 and PEBAX-5513 as the membrane matrix. A previous study showed that PEBAX-1657 showed a selectivity of 8.8 and permeance of 22.5 GPU [29]. In the case of the PEBAX-5513, the performance achieved a selectivity of 7.7 and permeance of 11.1 GPU [30]. Unfortunately, the separation performance achieved by the proportion of functional groups has not been yet clear. In this study, PEBAX 2533 was used as a new polymer matrix for facilitated olefin transport. PEBAX -2533 comprises of 80 wt % poly(tetramethylene oxide) (soft polyether blocks) and 20 wt % nylon-12 (hard polyamide blocks) [28]. Since polyether groups in polymer could show high permeability, this study was expected to find higher permeance than previous studies.

## 2. Materials and Methods 

### 2.1. Materials

Poly(ether-block-amide)-2533 (PEBAX-2533) was manufactured by Arkema Inc. Silver, while tetrafluoroborate (AgBF_4_, 98%) was purchased from TCI Fine Chemicals. Aluminum nitrate nonahydrate (Al(NO_3_)_3_·9H_2_O, ≥98%) was purchased from Aldrich Co. All chemicals were used as received without further purification.

### 2.2. Preparation of Membrane

The PEBAX-2533/Ag salt/Al(NO_3_)_3_ complex membrane was prepared using 3 wt % PEBAX-2533 solution. PEBAX-2533 was dissolved in a co-solvent with a 7:3 weight ratio of ethanol:water. Al(NO_3_)_3_·9H_2_O were added to the PEBAX-2533 solution in 0.1 molar ratio relative to Ag salt and stirred for 20 minutes. Ag salt was dissolved in ethanol at a weight ratio of 1: 9.33 to PEBAX-2533. Then, each solution was mixed and stirred for 10 minutes. The solutions were then coated on polysulfone microporous supports (Toray Chemical Korea Inc., South Korea) using an RK Control Coater (Model 202, Control Coater RK Print-Coat Instruments Ltd., UK). The composite membrane was placed in a vacuum oven and dried for at least 15 h.

### 2.3. Gas Separation Experiments

PEBAX-2533/metal salt/Al(NO_3_)_3_ complex membrane was tested under propane/propylene (50:50 vol %) mixed gas conditions. The gas permeation rate was measured using a bubble flow meter. The flow rate of the mixed gas was controlled by a mass flow controller (MFC). Gas chromatography (Young Lin 6500 GC system) was used to measure propane/propylene selectivity. The unit of gas permeance is GPU, where 1 GPU = 1 × 10^−6^ cm^3^ (STP)/(cm^2^s cmHg).

### 2.4. Characterization

The cross section of the composite membrane was confirmed using scanning electron microscopy (SEM, JEOL JSM-5600LV). Raman spectra were collected using a Bruker Optics Ram II Raman module with a resolution of 4 cm^−1^. The IR peak shift was measured by a VERTEX 70 Fourier transform infrared (FTIR) spectrometer; 32 scans were signal-averaged with a resolution of 4 cm^−1^. The thermal stability of the membranes was confirmed by using thermogravimetric analysis (TGA; Universal V4.5 A, TA Instruments).

## 3. Results

### 3.1. SEM

**Figure 1:**
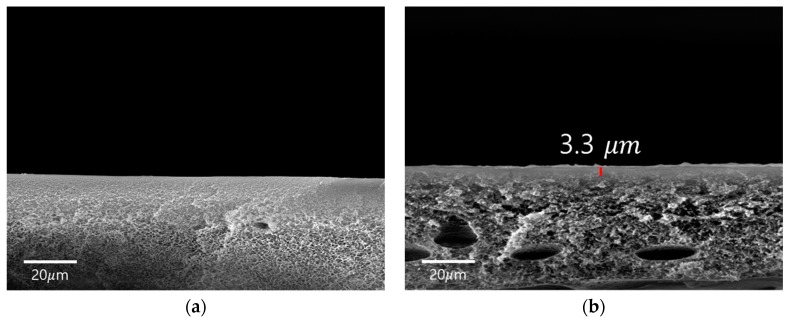
SEM images of (**a**) neat porous polymer support and (**b**) PEBAX-2533/metal salt/Al salt membrane coated on porous polymer support.

The SEM image showed the cross section of the membrane. As shown in Figure 1, PEBAX-2533/metal salt/Al salt solution was coated on the porous polysulfone support. The thickness of the selective layer was about 3.3 μm. The structure of the polymer support was observed as being sponge-like and the shape remained constant after coating the membrane solution.

### 3.2. FT-IR

**Figure 2:**
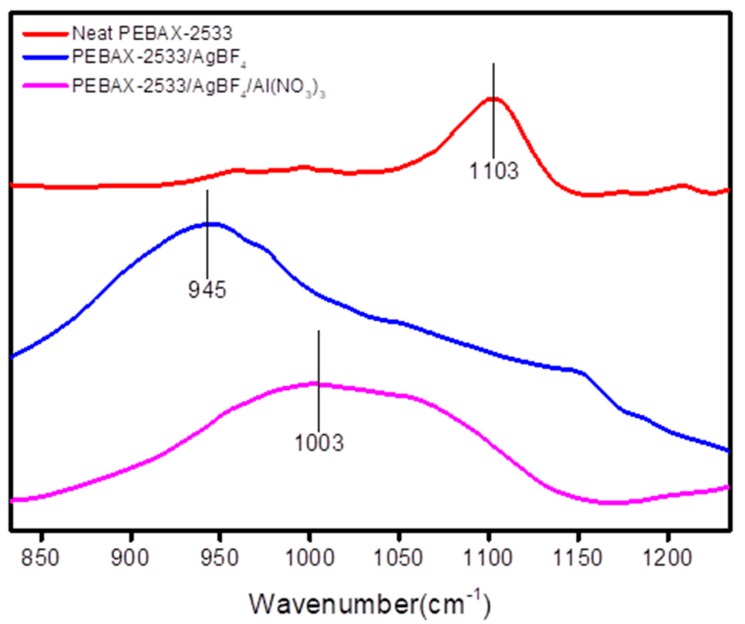
FT-IR spectra of the ether group of neat PEBAX-2533, PEBAX-2533/metal salt and PEBAX-2533/metal salt/Al salt.

**Figure 3:**
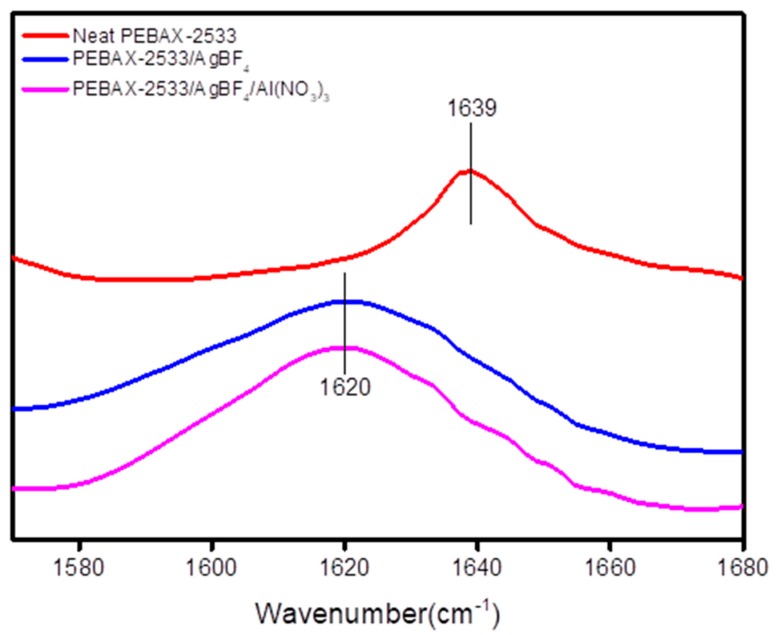
FT-IR spectra of carbonyl group peaks of neat PEBAX-2533, PEBAX-2533/metal salt and PEBAX-2533/metal salt/Al salt.

The interaction between Ag cations and the functional groups such as the ether group and amide group in PEBAX polymer chains was analyzed through infrared spectroscopy. Figure 2 shows that C–O stretching bonds of PEBAX-2533 and the free C–O bonds of the neat PEBAX-2533 were observed at 1103 cm^−1^. After incorporation of the Ag salt, the C–O bond shifted from 1103 to 945 cm^−1^. This shift was generated by the weakening of the C–O bond with donating electrons from the C–O bond to metal ions. When Al salt was added, the C–O peak shifted to 1003 cm^−1^. As a result of the interaction of the NO_3_^−^ of Al salts with Ag ions, the strength of C–O bonds increased. The C=O stretching bonds of amide group is shown in Figure 3. The stretching bond of the carbonyl group shifted from 1639 (observed in neat PEBAX) to 1620 cm^−1^. However, unlike ether group, there was almost no change of peak when Al salts were added. This indicated that Ag ions that interacted with the C=O bond did not interact with NO_3_^−^. Thus, most of the NO_3_^−^ was shown to interact with the ether group, which accounts for a large part of PEBAX-2533.

### 3.3. Separation Performance

**Figure 4:**
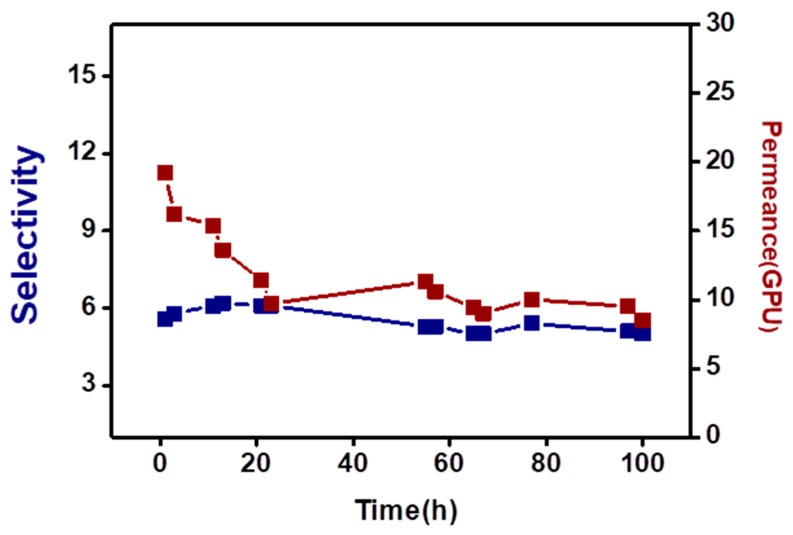
Gas separation performance of PEBAX-2533/metal salt/Al salt composite membrane over time.

**Scheme 1:**
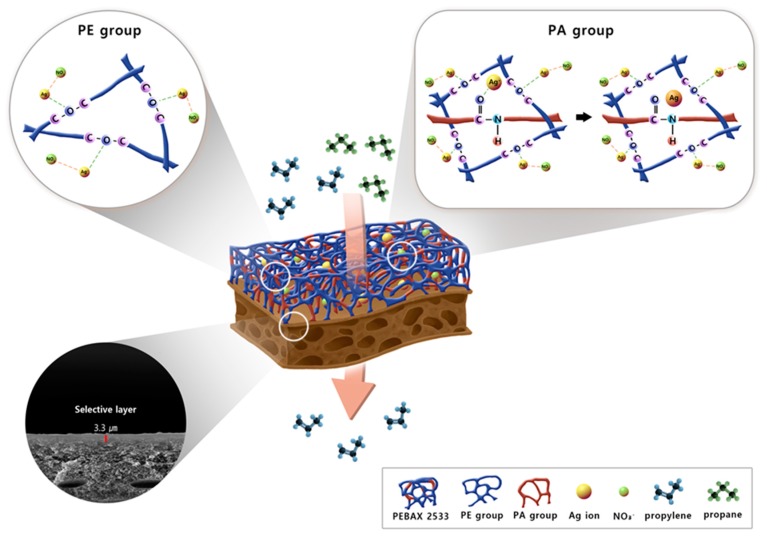
Facilitated propylene transport in PEBAX-2533/metal salt/Al salt.

Figure 4 shows the gas permeation test of PEBAX-2533/metal salt/Al salt, which facilitated transport membrane for more than 100 h. The selectivity rating of propylene/propane mixture remained steady at about 5. On the other hand, the permeance decreased from initial 15 to 10 GPU after 20 h. These decreases of permeance could be described as being the elimination of the remaining water and ethanol in membranes to reduce the polymer’s flexibility, thereby decreasing the membrane’s permeance. In a previous experiment, the permeation performance of the PEBAX-1657/metal salt/Al salt and The PEBAX-5513/metal salt/Al salt showed selectivity of 8.8 with 22.5 GPU, and selectivity of 7.7 with 11.1 GPU, respectively. [26,27]. Compared to the previous results, the PEBAX-2533/metal salt/Al salt composite membrane had a relatively low performance. Note that amide groups had better facilitative effects than ether groups due to their polarizing effect on metal nanoparticles. However, in the case of PEBAX-2533, ether groups had a high proportion of 80%, resulting in the low effect of amide groups on separation performance. Scheme 1 shows the facilitated propylene transport in PEBAX-2533/metal salt/Al salt.

### 3.4. RAMAN

**Figure 5:**
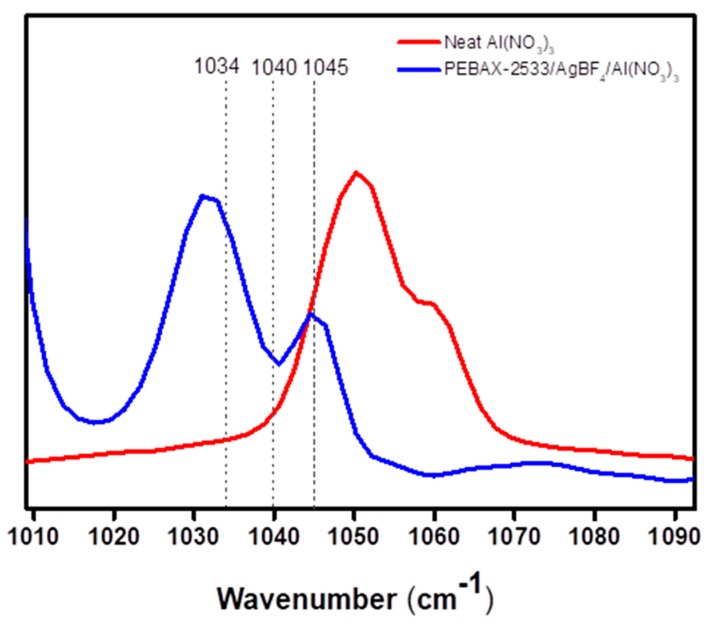
Raman spectra of NO_3_^−^ ions in neat Al salt and PEBAX-2533/metal salt/Al salt complex.

Figure 5 displays raman spectra used to investigate the state of NO_3_^−^ of Al salts. The state of NO_3_^−^ ions is shown in Figure 4, which shows the free ions (1034 cm^−1^), ion pairs (1040 cm^−1^) and ion aggregates (1045 cm^−1^). NO_3_^−^ in neat Al salts existed almost in an ion aggregates state at 1055 cm^−1^. In PEBAX-2533/metal salt/Al salt complex, the ion state of NO_3_^−^ was appeared at 1031 and 1044 cm^−1^. These results verified that NO_3_^−^ ions existed mostly as free ions. Thus, abundant free NO_3_^−^ ions from countercation Al^3+^ could easily interact with metal ions, preventing any reduction to metal nanoparticles.

### 3.5. TGA

**Figure 6:**
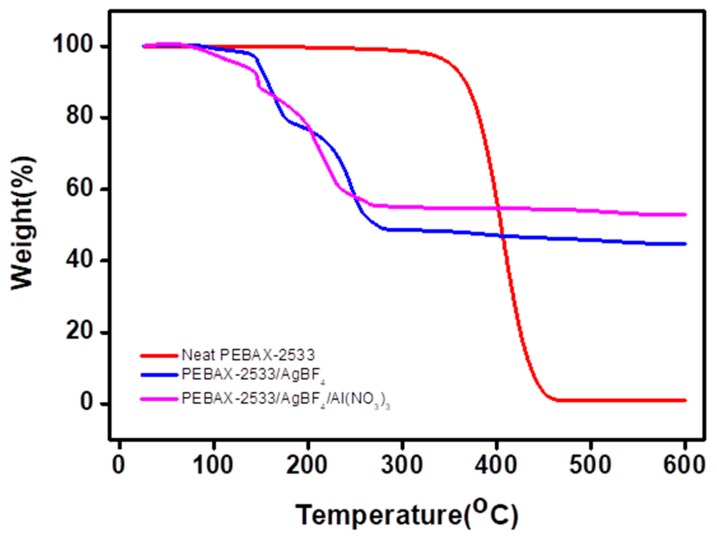
TGA curves for neat PEBAX-2533, PEBAX-2533/AgBF4 and PEBAX-2533/metal salt/Al salt complex.

Thermal analysis of PEBAX-2533, PEBAX-2533/metal salt and PEBAX-2533/metal salt/Al salt composite membranes were confirmed by TGA of the room temperature to reach 600 °C. As seen in Figure 6, pure PEBAX-2533 lost weight once at 330 °C. The high thermal stability of PEBAX-2533 was due to the intermolecular hydrogen bonding. When metal salts were added, the thermal stability was reduced at low temperatures. This weakening was thought to be because the incorporated metal ions prevented intermolecular interactions by interacting with amide and ether groups in the polymer. Similarly, when Al salts were added, the intermolecular interactions were disturbed, leading to plasticization of the polymer. However, Figure 5 demonstrates that after 400 °C, the polymer was more cross-linked than neat PEBAX-2533. The increase in thermal stability could be attributed to the transient crosslinking effect of metal ions and generated metals.

## 4. Conclusions

Higher permeance was expected when using PEBAX-2533 with a higher ether ratio compared to the PEBAX-1657 or PEBAX-5513 utilized as a polymer matrix in previous studies. However, the permeance of the membrane unexpectedly did not improve, and the selectivity was reduced due to the decrease in the ratio of the amide group, which was the selective block of PEBAX. Furthermore, even though Al salts were known to stabilize the Ag ions, it was confirmed by using FT-IR that Al salts did not stabilize the metal ions interacting with the amide group due to the high ether group ratio in PEBAX-2533. Thus, higher ether groups in the copolymer matrix could interfere with the stabilizing effect of Al salts on metal ions in the polymer/metal salt/Al salt complex membrane, as shown in Figure 1. A study of the performance change based on the copolymer ratio of the ether group and amide group in block-copolymer is anticipated to be helpful for the design of polymer structures for facilitated olefin transport membrane used in the petroleum industrial field.
